# Plasmapheresis for Spur Cell Anemia in a Patient with Alcoholic Liver Cirrhosis

**DOI:** 10.1155/2018/9513946

**Published:** 2018-06-21

**Authors:** Kenji Miki, Takashi Maruki, Shinsaku Imashuku

**Affiliations:** ^1^Department of Internal Medicine, Uji-Tokushukai Medical Center, Uji 611-0042, Japan; ^2^Department of Laboratory Medicine, Uji-Tokushukai Medical Center, Uji 611-0042, Japan

## Abstract

**Background:**

Spur cell anemia (SCA) is a cause of hemolytic anemia in patients with alcoholic liver cirrhosis. Because dyslipidemia is related to the development of spur cells, SCA was previously treated with plasmapheresis.

**Case Report:**

A 52-year-old Japanese man with SCA associated with alcoholic liver cirrhosis (Child–Pugh C) underwent two rounds of plasmapheresis. Clinical features and serum lipid concentrations were compared before and after plasmapheresis. Although indirect hyperbilirubinemia and SCA persisted after plasmapheresis, reticulocyte counts significantly decreased from 22.4% to 4.5%, and Hb levels improved without red cell transfusions. Analysis of lipids showed that total and free cholesterol, HDL cholesterol, phospholipid, and apo-AI concentrations, all of which were reduced before plasmapheresis, had improved after treatment, while LDL cholesterol, lipoprotein (a), and apo-AII concentrations, which were also reduced before plasmapheresis, remained unchanged.

**Conclusions:**

Despite plasmapheresis partially ameliorating the degree of hemolysis, the persistence of SCA may have been linked with the lack of improvement in certain types of lipid metabolism.

## 1. Introduction

The pathogenesis of anemia in liver cirrhosis is complex and multifactorial and can involve factors such as portal hypertension-induced sequestration, alterations in iron metabolism, bone marrow damage, and/or increased blood loss due to hemorrhage and/or hemolysis [1]. Spur cell anemia (SCA) is an acquired hemolytic anemia, characterized by an increase in the percentage of spur cells (acanthocytes) associated with liver cirrhosis [2]. The presence of ≥5% spur cells on peripheral blood smears has been observed in 31% of patients with liver cirrhosis [3]. Patients with SCA are generally alcohol abusers with decompensated cirrhosis, who show hemolysis and frequently, hemochromatosis [4–7]. Their symptoms may include severe jaundice and coagulopathy, with or without encephalopathy, and they may require frequent red blood cell (RBC) transfusions. Dyslipidemia is involved in the pathogenesis of SCA [8–11], and SCA is an independent predictor of mortality in patients with liver cirrhosis [3]. This report describes a cirrhotic patient with SCA who was treated with two courses of plasmapheresis.

## 2. Case Report

**Figure 1:**
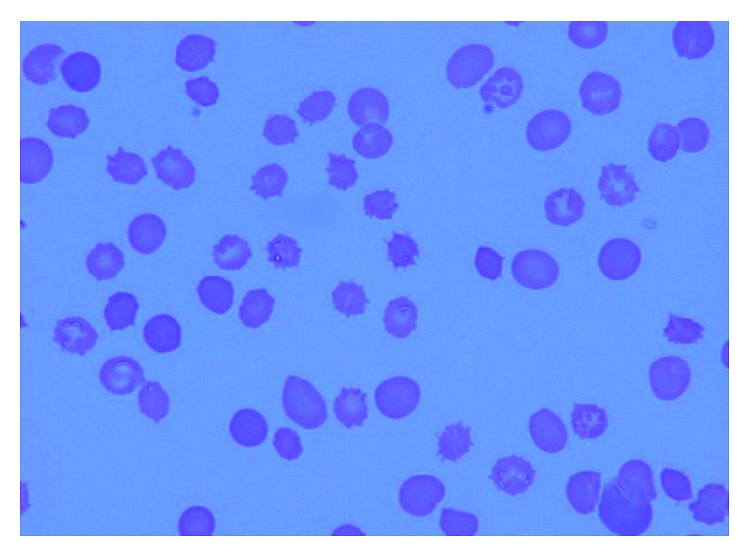
Peripheral blood fresh smear of the patient. Spur cells were observed in approximately 25% of red blood cells.

A 52-year-old Japanese man with alcoholic liver cirrhosis and a history of previous esophageal varices and hepatic encephalopathy was referred to our hospital. At admission, he was afebrile, icteric, and anemic and complained of abdominal pain due to accumulated ascites. He had none of the neurological symptoms of neuroacanthocytosis [12]. He was alert, with a blood pressure of 126/66 mmHg, a heart rate of 108/min, a respiratory rate of 20/min, and an SpO_2_ of 95% (room air). Laboratory data are shown in Table 1. He was found to have pleural fluid, ascites associated with liver cirrhosis (Child–Pugh C with 12 points) and hypoalbuminemia, and chronic kidney dysfunction. His indirect bilirubin concentration and reticulocyte counts were increased, and his haptoglobin concentration was decreased, while his vitamin B12 and folate levels were normal. A blood smear showed spur cells rather than fragmented red cells (Table 1, Figure 1). Assessments of serum lipid concentrations showed markedly reduced triglyceride, HDL cholesterol, LDL cholesterol, lipoprotein (a), phospholipid, apo-AI, and apo-AII concentrations (Table 2). Mild splenomegaly was noted. He was slightly diabetic with high serum glycoalbumin (21.5%; reference 11.6–16.4%) and showed coagulopathy, including thrombocytopenia, prolonged APTT, and hypofibrinogenemia, as well as reduced AT-III. Assessments of coagulation factors showed that factor II, V, VII, and IX activities were all reduced, while factor VIII was not. ADAMTS13 activity was within the normal range (Table 1). Blood smear preparations after incubating the patient's serum with control RBCs of the same blood type for 24 hours clearly showed formation of spur cells, indicating that this patient's dyslipidemia was responsible for spiculated control RBCs (data not shown). The patient was diagnosed with SCA.

He had been drinking prior to his admission; however, during his stay in the hospital for 4 weeks, he was in a state of complete abstinence. After admission, he was first treated with ascites drainage, RBC transfusions, occasional albumin, and FFP infusions. Thereafter, two courses of plasmapheresis (2,400 mL/course) were performed over 3 weeks' duration.

### 2.1. Effects of Plasmapheresis on Serum Lipids and Clinical Data

The patient's reticulocyte counts decreased from 22.4% before to 4.5% after plasmapheresis. He also experienced increases in concentrations of total cholesterol, from 121 mg/dL to 214 mg/dL; phospholipids, from 112 mg/dL to 197 mg/dL; and apo-AI, from 48 mg/dL to 89 mg/dL. However, his lipoprotein (a), LDL cholesterol, and apo-AII concentrations, all of which were low before plasmapheresis, remained unchanged after, and his serum concentrations of indirect bilirubin and LDH did not improve significantly (Table 2). Plasma fibrinogen concentrations improved rapidly after each course of plasmapheresis, but gradually declined to subnormal levels. The percentage of spur cells was unchanged or increased slightly. The patient's general condition gradually improved with the disappearance of pleural fluid and ascites, and Hb concentration >8.0 g/dL without RBC transfusions. He was subsequently able to be discharged.

## 3. Discussion

Patients with alcoholic cirrhosis may have “target” or “spur” red cells and hemolytic anemia [2, 8]. Since the initial description of SCA in the 1960s [2], there have been several sporadic case reports. Scanning electron microscopy of RBCs, which clearly demonstrates spur cells (acanthocytes) with multiple spicules irregularly distributed over the RBC surface, has been reported to be useful in the diagnosis of SCA [7].

In general, excessive alcohol consumption results in dysregulation of lipid metabolism [13–16]. Patients with alcoholic cirrhosis and Child–Pugh C stage show significant reductions in serum concentrations of total, HDL, and LDL cholesterol, but not of triglycerides [14, 15]. Low serum lipoprotein (a) concentrations have also been reported in patients with liver cirrhosis [13]. However, the precise mechanisms relating dyslipidemia with the development of SCA remain unknown. Marked decreases in serum concentrations of cholesterol, triglycerides, and phospholipids have been reported [10], as well as increased serum concentrations of free cholesterol [8]. Markedly reduced plasma concentrations of apo-AI, apo-AII, and HDL3 have also been reported in patients with alcoholic cirrhosis and SCA [9, 11]. The development of SCA in cirrhotic patients has been associated with changes in cholesterol content of RBC membranes [8]. Cholesterol transfer induced by all lipoprotein fractions in sera may accompany reductions in lecithin cholesterol acyltransferase (LCAT) activity, thereby affecting cellular cholesterol efflux [8]. This may result in a striking increase in cholesterol content of spur cells and a high cholesterol/phospholipid ratio in RBC membranes [8]. Although we could not analyze the lipids in RBC membranes of our patient, we compared pre- and postplasmapheresis lipid abnormalities in relation to SCA. Before plasmapheresis, this patient showed lipid and lipoprotein abnormalities as previously described, whereas, after plasmapheresis, his low lipoprotein (a), LDL cholesterol, and apo-AII concentrations remained unchanged, accompanied by persistent indirect hyperbilirubinemia and SCA. It remains unknown how reduced serum lipoprotein (a), LDL cholesterol, and apo-AII played a role in persistent SCA [17, 18].

Treatment of SCA has been disappointing and usually indicates end-stage liver disease. The importance of abstinence from alcohol has been emphasized as the mainstay of management; however, success is rare. Formerly, patient treatments included splenectomy and frequent transfusions [19]. Plasmapheresis, first described in Japan for patients with SCA, was temporarily effective but resulted in eventual poor outcome [20–22]. Indeed, as many as ten courses of plasmapheresis were unable to reverse SCA [20]. At present, liver transplantation is regarded as the most effective treatment [5–7], with reversibility of SCA observed only after liver transplantation [5, 23]. Unfortunately, our patient was ineligible for liver transplantation, because of no available donors and that he repeatedly failed to abstain from alcohol. However, we assessed the effects of plasmapheresis on the pattern of dyslipidemia as well as on variable indicators of SCA, since previous reports on plasmapheresis did not analyze its effects on dyslipidemia in detail [20–22]. Our results may provide useful information for future studies on SCA.

In summary, a precise understanding of the correlation of dyslipidemia and SCA remains elusive and more studies are necessary. In addition, further research is required to determine specific pharmaceutical treatments on SCA-related dyslipidemia for patients with liver cirrhosis who are ineligible for liver transplantation.

## Figures and Tables

**Table 1:** Laboratory data.

*CBC*	
WBC (3,000–8,500)/*µ*L	8,300
Hb (13–17) g/dL	7.9
MCV (83–100) fL	118.5
PLTs (150–360 K)/*µ*L	33 K

*Serum chemistry*	
Total protein (6.7–8.3) g/dL	5.9
Albumin (4.1–5.2) g/dL	2.3
LDH (122–228) U/L	438
GGTP (12–49) U/L	19
Total bilirubin (0.2–1.3) mg/dL	15.65
Direct bilirubin (0.1–0.3) mg/dL	8.0
Ammonia (20–70) *µ*g/dL	95
Ferritin (39–340) ng/mL	344
BUN (7.8–18.9) mg/dL	35.1
Creatinine (0.64–1.11) mg/dL	1.92
Vitamin B12 (260–1,050) pg/mL	3,240
Folate (4.4–13.7) ng/mL	6.5
Glycoalbumin (11.6–16.4) %	21.5

*Hemolysis-related*	
Reticulocytes (0.3–1.1) %	22.4
Haptoglobin (58–160) mg/dL	3
Coombs test	
Direct/indirect	Neg/neg

*Coagulation*	
APTT (control 27.5) sec	>69.1
PT-INR	1.65
Fibrinogen (200–400) mg/dL	78
AT-III (23.6–33.5) mg/dL	8.8
FDP (0.0–2.5) *µ*g/mL	14.2
Factor II (66–118) %	31.9
FV (73–122) %	29.0
FVII (54–162) %	22.4
FVIII (78–165) %	89.0
FIX (67–152) %	33.1
ADAMTS13 (70–120) %	71.8

**Table 2:** Comparison of serum lipids between before and after plasmapheresis.

Lipids	2018/03/15		2018/04/06
Triglyceride (33–149) mg/dL	53		68
Total cholesterol (130–219) mg/dL	121		214
HDL cholesterol (40–70) mg/dL	15		30
LDL cholesterol (63–139) mg/dL	29		26
Free cholesterol (25–60) mg/dL	54		101
Phospholipid (PL; 150–250) mg/dL	112		197
Total cholesterol/PL	1.08		1.08
Free cholesterol/PL	0.45	PPx2	0.47
Lipoprotein (a) (∼40) mg/dL	4.1		3.3
Β-lipoprotein (150–600) mg/dL	185		NT
Apo-AI (119–155) mg/dL	48		89
Apo-AII (25.9–35.7) mg/dL	5.2		7.2
Phospholipase A2 (130–400) mg/dL	329		NT

Others			
Reticulocyte counts (%)	22.4		4.5
Hb (g/dL)	7.9		9.1
LDH (U/L)	438		380
Indirect bilirubin (mg/dL)	7.65		14.7
Haptoglobin (g/dL)	3		2
Spur cells (%)	25		35

PP, plasmapheresis; Hb, hemoglobin; LDH, lactate dehydrogenase.
